# Systematic review of cardiovascular neurocristopathy—contemporary insights and future perspectives

**DOI:** 10.3389/fcvm.2024.1333265

**Published:** 2024-04-09

**Authors:** Osama Soliman, Yogesh Acharya, Martine Gilard, Garry Duffy, William Wijns, Venkatesh Kannan, Sherif Sultan

**Affiliations:** ^1^ Department of Cardiology, Galway University Hospital, Galway, Ireland; ^2^ CORRIB-CURAM-Vascular Group Collaborators, University of Galway, Galway, Ireland; ^3^ Western Vascular Institute, Department of Vascular and Endovascular Surgery, University Hospital Galway, University of Galway, Galway, Ireland; ^4^ Department of Cardiology, La Cavale Blanche Hospital, Brest, France; ^5^ Anatomy and Regenerative Medicine Institute (REMEDI), School of Medicine, University of Galway, Galway, Ireland; ^6^ Irish Centre for High-End Computing (ICHEC), University of Galway, Galway, Ireland; ^7^ Department of Vascular Surgery and Endovascular Surgery, Galway Clinic, Doughiska, Royal College of Surgeons in Ireland and University of Galway Affiliated Hospital, Galway, Ireland

**Keywords:** neural crest cells, cardiac neural crest cells, cardiovascular neurocristopathy, cardiac neurocristopathy, vascular neurocristopathy, aortopathy, bicuspid aortic valve

## Abstract

**Introduction:**

Neural crest cells (NCCs) are multipotent and are attributed to the combination of complex multimodal gene regulatory mechanisms. Cardiac neural crest (CNC) cells, originating from the dorsal neural tube, are pivotal architects of the cardio-neuro-vascular domain, which orchestrates the embryogenesis of critical cardiac and vascular structures. Remarkably, while the scientific community compiled a comprehensive inventory of neural crest derivatives by the early 1980s, our understanding of the CNC's role in various cardiovascular disease processes still needs to be explored. This review delves into the differentiation of NCC, specifically the CNC cells, and explores the diverse facets of non-syndromic cardiovascular neurocristopathies.

**Methods:**

A systematic review was conducted as per the PRISMA Statement. Three prominent databases, PubMed, Scopus, and Embase, were searched, which yielded 1,840 studies. We excluded 1,796 studies, and the final selection of 44 studies formed the basis of this comprehensive review.

**Results:**

Neurocristopathies are a group of genetic disorders that affect the development of cells derived from the NC. Cardiovascular neurocristopathy, i.e., cardiopathy and vasculopathy, associated with the NCC could occur in the form of (1) cardiac septation disorders, mainly the aortico-pulmonary septum; (2) great vessels and vascular disorders; (3) myocardial dysfunction; and (4) a combination of all three phenotypes. This could result from abnormalities in NCC migration, differentiation, or proliferation leading to structural abnormalities and are attributed to genetic, familial, sporadic or acquired causes.

**Discussion:**

Phenotypic characteristics of cardiovascular neurocristopathies, such as bicuspid aortic valve and thoracic aortic aneurysm, share a common embryonic origin and are surprisingly prevalent in the general population, necessitating further research to identify the underlying pathogenic and genetic factors responsible for these cardiac anomalies. Such discoveries are essential for enhancing diagnostic screening and refining therapeutic interventions, ultimately improving the lives of individuals affected by these conditions.

## 1 Introduction

The cellular events during embryonic development unveils the remarkable complexity of life within which a particular group of cells, known as the neural crest cells (NCC), holds a pivotal role (1–3). Neurocristopathy a term that encompasses disorders within these NCC (1, 2, 4).

The cardiac neural crest (CNC) cells are specialized entities that assume a commanding role in the cardiovascular development of aortic arch arteries and the cardiac outflow tract (5, 6). The two interrelated conditions—cardiovascular neurocristopathy and NC aortopathy, usually manifest as a bicuspid aortic valve (BAV) (7, 8). BAV is marked by aortic dilation and/or acute aortic dissection (8–10).

The true magnitude of this aortopathy remains veiled, but recent revelations hint at its profound impact (11). While the scientific community once crafted an inventory of NC derivatives, it was not until the early 1980s that the heart revealed its secret—within the vagal NC lies a distinct and dynamic entity, the CNC (12). This population of cells not only shapes the cardiovascular system but also orchestrates the birth of the thymus, the thyroid glands, and the cardiac ganglia (3).

This systematic review is to unravel the intricate differentiation within NCC, with a specific focus on CNC cells. Our quest takes us into non-syndromic cardiovascular neurocristopathies, particularly within the young population, a vulnerable and often overlooked cohort.

## 2 We illuminate three crucial facets

1. The non-syndromic cardiovascular neurocristopathy, explores a myriad of phenotypes, from NC aortopathy to NC BAV and congenital heart disease (CHD).
2. We inspect the cardiovascular pathologies arising from disruptions in regulatory factors, and we spotlight the profound importance of various signalling pathways that thread through embryonic development.
3. We examine its connections to cerebro-vascular phenomena, offering insights into the mechanisms underpinning associated abnormalities. In doing so, we also explore potential therapeutic advancements.

## 3 Materials and methods

### 3.1 Selection strategy

To conduct a comprehensive review, we executed a systematic literature search with a sharp focus on our study objectives. Three prominent databases, PubMed, Scopus, and Embase, served as our sources of information. Our search revolved around the overarching theme of cardiovascular neurocristopathy and its various manifestations, using specific keywords such as “cardiovascular neurocristopathy,” “neural crest aortopathy,” “neural crest bicuspid aortic valve,” “neural crest heart disease,” and “non-syndromic aortopathy or neurocristopathy.” We restricted our search to articles published in the English language from the inception of each database until March 2023.

It is crucial to note that cardiovascular neurocristopathy encompasses both syndromic and non-syndromic cases. However, for this manuscript, our focus remained solely on non-syndromic neurocristopathy. This decision led to the exclusion of articles that discussed acquired causes and multi-organ involvement associated with syndromic neurocristopathy.

## 4 Results

### 4.1 Selection criteria

Our selection criteria adhered to the guidelines set by the PRISMA Statement (13). The initial search sought to map the existing literature concerning cardiovascular neurocristopathy, NC aortopathy, BAV and CHD. Subsequently, our search was fine-tuned to target the non-syndromic subset and the young-age population, defined as individuals between the ages of 18 and 65 as per WHO guidelines. The specific focus remained on non-syndromic NC aortic conditions and associated myocardial dysfunction, prompting the exclusion of articles dealing with syndromes, acquired causes, or multiorgan involvement.

Our diligent search yielded a total of 1,840 articles. Following a meticulous review process, 1,796 research articles were excluded, taking into account duplications and alignment with our inclusion criteria. This stringent process culminated in a final selection of 44 articles that form the basis of this comprehensive review.

### 4.2 Quality assessment and data collection

This review is anchored in original full-text research articles and review papers. The thoroughness of our approach was maintained by vigilant scrutiny to eliminate duplications. The abstracts of these articles underwent rigorous analysis to ensure their quality and relevance to our study objectives. Subsequently, each research paper was meticulously evaluated to gauge its suitability for inclusion in this review.

**Figure 1:**
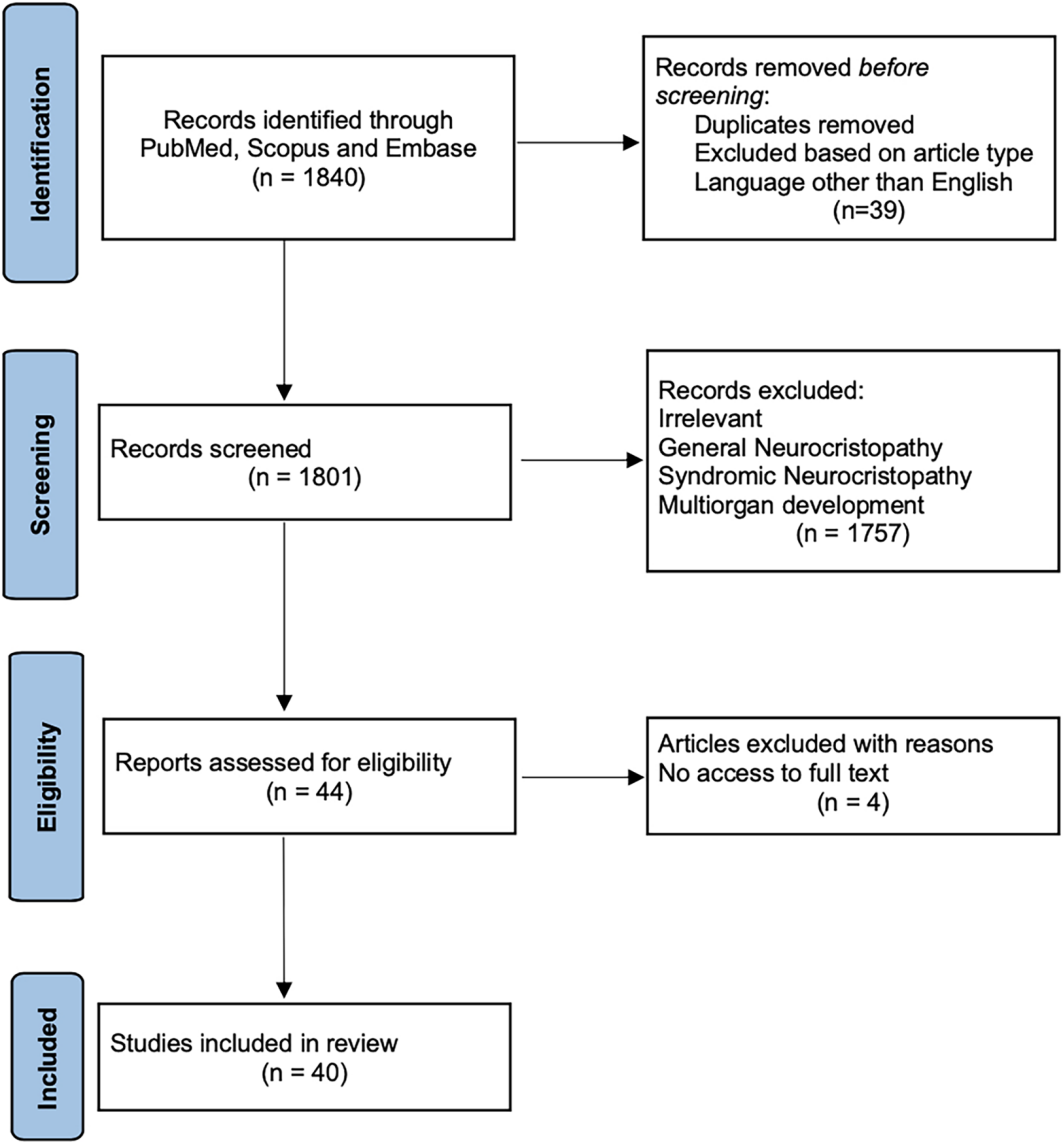
PRISMA flow diagram. This graph serves as a visual representation of the literature inclusion and exclusion at each stage, in accordance with the PRISMA Statement, guiding our systematic and methodical approach in conducting this comprehensive review.

Following the removal of duplicate records, an additional four articles were excluded due to a lack of full-text availability. Ultimately, 40 articles were included in this final review paper, aligning with our objectives, which is outlined in the given PRISMA flow diagram (Figure 1).

### 4.3 Embryological origin of vascular tree

**Figure 2:**
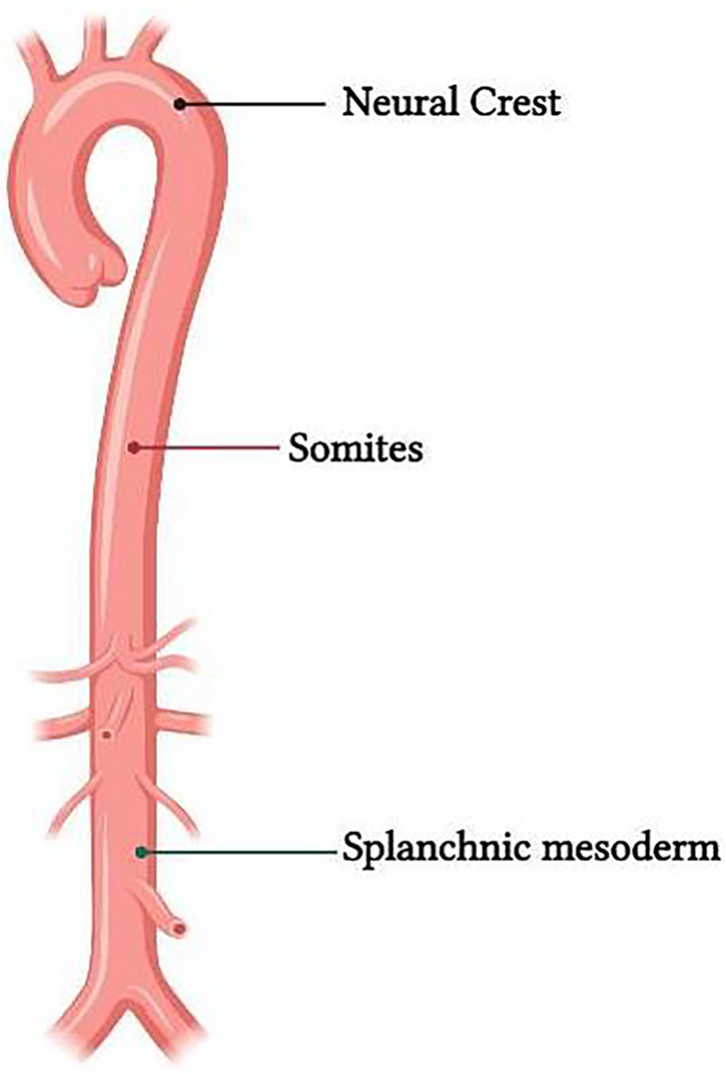
Representation of the difference in embryological origin of the thoracic and abdominal aorta.

Vascular tree has a variable embryonic origin (Figure 2) (14–16). Embryonic fate-mapping studies have demonstrated that the aortic root, ascending aorta and aortic arch are populated by vascular smooth muscle cells (VSMCs) arising from NC, whereas VSMCs from the paraxial mesoderm or somites populate the descending aorta (17, 18). Studies have shown that CNS cells also contribute to the VSMCs found in some of the branchial arch arteries (19).

### 4.4 Neural crest cells (NCC)

NCC originate from the neuroepithelium, i.e., the exterior layer or ectoderm, which is responsible for forming the neural tube that eventually becomes the central nervous system (3, 20). Gastrulation causes mesodermal structures to develop between the endoderm and ectoderm. The paraxial mesoderm divides into segmented groups of cells known as somites, which are found on either side of the neural tube. Although these somites are only temporary, they can produce a wide range of tissues and organs (21).

**Figure 3:**
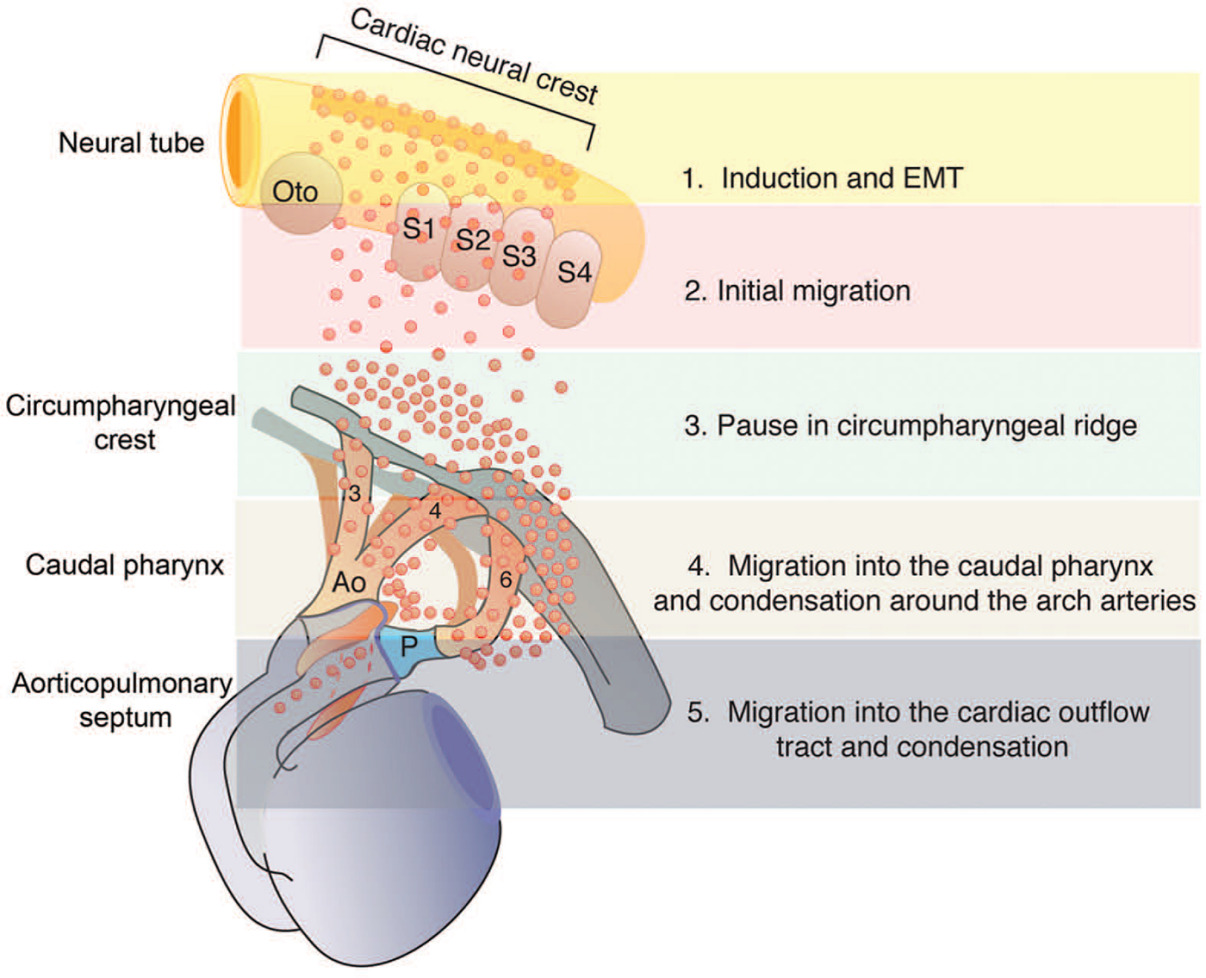
The migration and dispersion of cardiac neural crest cells (NCC). This graph displays the migration and dispersion of cardiac NCC from the neural tube to the caudal pharynx and then to the outflow tract (OFT). Reprinted with permission from Kirby et al. (22).

During the early stages of embryonic development, the NC exists as a narrow band of cells located between the neural and non-neural ectoderm (3, 20). The NCCs migrate dorsally and then delaminate in a rostrocaudal pattern as the neural tube shuts (Figure 3). The highly multipotent and temporarily migratory NC is a vertebrate-specific cell population divided into four main subpopulations based on their migratory path, terminal location, and differential abilities: cranial, vagal, trunk, and sacral (22).

### 4.5 Cardiac neural crest (CNC) cells

Studies have indicated that the vagal NC consists of a smaller specified group of cells termed as the CNC, known to significantly contribute to cardiovascular development, along with aiding in the development of the thymus, thyroid glands, and cardiac ganglia (3, 23). CNC cells develop along the neural tube from the mid-diencephalon to the embryo's most caudal extremity. These crest cells are classified as cranial or trunk based on their ability to generate ecto-mesenchyme (22).

CNC cells have an impact on heart formation both directly and indirectly (6, 11) (Table 1). Alignment and outflow septation are two distinct phases that co-occur simultaneously during cardiac morphogenesis. In the alignment phase, the cardiac tube aligns with the developing vasculature, while in the outflow septation phase, the ventricular outflow tract is divided into the aorta and pulmonary artery. Also, CNC cells are necessary for correct looping and extension of the heart tube, including early myocardial function, and are crucial in the formation of the outflow septum and arch artery patterning (22, 24).

**Table 1:** Table showing the cardiovascular neural crest cells (NCC) derivatives.

NCC Derivatives	Description
Outflow septation & aortic arch derivatives (24)	NCC contributes to the formation of pharyngeal arches and derivatives, along with aortico-pulmonary outflow septum formation.
Cardiac valves (6, 25, 26)	Cardiac neural crest (CNC) cells are associated with the formation of the semilunar valves (25) and atrioventricular (AV) valves (26).
Myocardial cells (27–29)	CNC cells are associated with cardiomyogenesis (27), cardiomyocytes and heart regeneration (28) in Zebrafish, and their loss could lead to adult-onset hypertrophic cardiomyopathy (29).
Conductive system (30, 31)	CNC cells contribute to the formation of the conduction system in the heart.
Pericytes (32–34)	CNC cells lead to the formation of all blood vessels of the face and forebrain (32), the central nervous system (34), and vascular smooth muscle cells of the Brain (33).
Excitation coupling and C2+ handling (35, 36)	CNC cells are associated with L-type calcium current in the heart with persistent truncus arteriosus.

### 4.6 Cellular mechanism of NC differentiation

**Figure 4:**
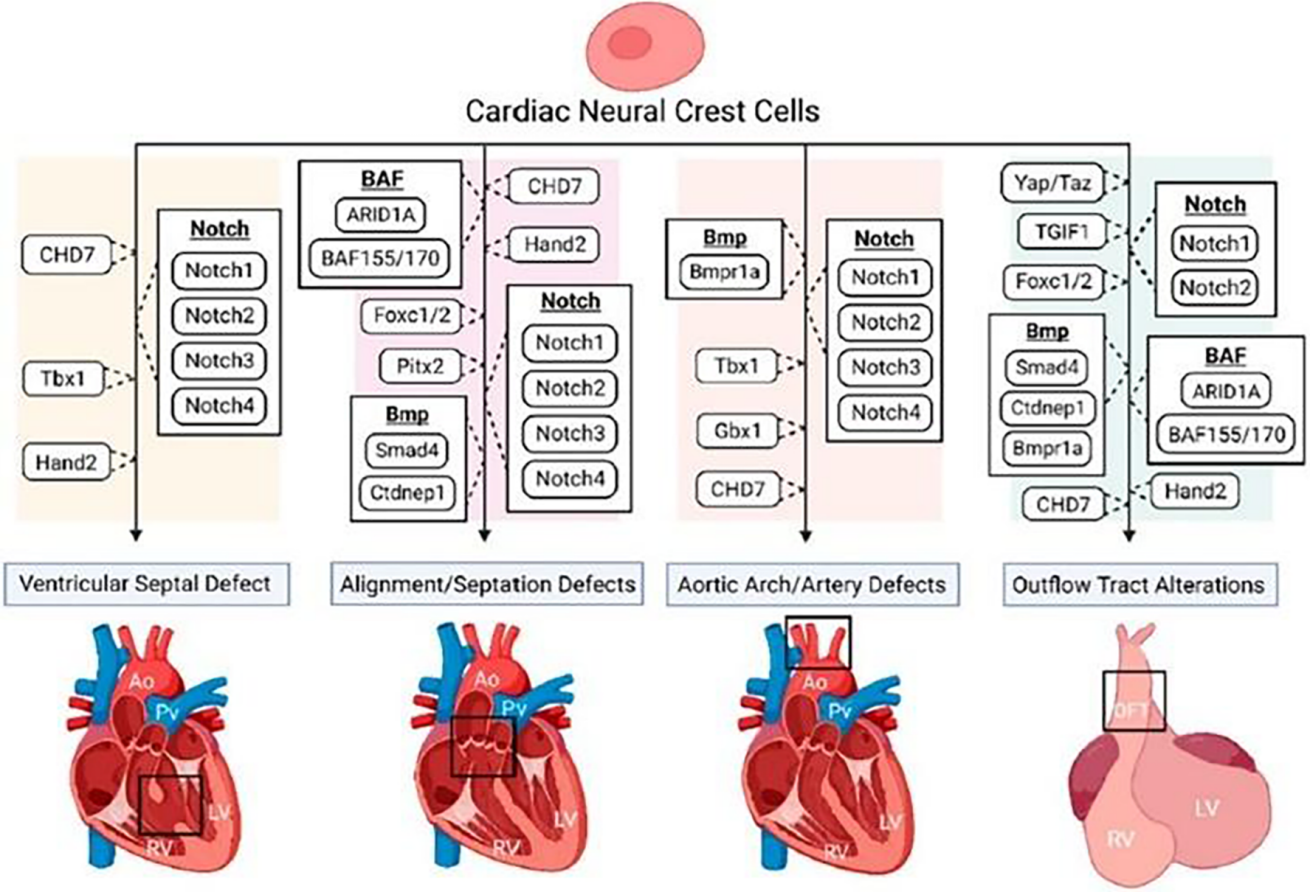
The disruption of various regulatory pathways of cardiac neural crest (CNC) cells. This figure shows the role of CNC cells in numerous congenital heart defect phenotypes. Reprinted with permission from Erhardt et al. (36).

During gastrulation, CNC cells are induced from the dorsal region of the neural plate border, orchestrated by various regulatory pathways such as Bmp, Wnt, Notch, and, more recently recognized, Hippo (Figure 4) (37).

NC progenitors obtain their migratory potential by undergoing epithelial-to-mesenchymal transition (EMT) (38). It has been demonstrated that different signaling pathways, such as BMP, WNT, and retinoic acid, play critical roles in regulating NC EMT and migration for proper cardiovascular development. However, more research into the crosstalk between signalling networks is required. Delamination from the neural tube is regulated via BMP-dependent Wnt1 activity, with Wnt1 expression turning off soon after the cells leave the neural tube (39). Many transcription factors and signalling molecules have been implicated in the later migration steps, proliferation, survival, and differentiation of the CNC.

Studies have also implicated the Notch signalling pathway for healthy NC development, including cellular proliferation and specification (40). Mutations in Notch-target genes such as Notch1 and Notch2, as well as Notch ligands such as Jagged1, have been demonstrated to produce a range of heart abnormalities in mice, including VSD and malformations of the cardiac OFT and great arteries (41–43).

Varadkar and colleagues (43) discovered that Notch2 is required for proper NC-derived aortic and pulmonary smooth muscle formation and disrupting Notch2 in post-migratory CNC cells with the Pax3-Cre driver results in narrowed OFT and OFT arteries (aorta and pulmonary) in E17.5 mouse embryos, indicating a cell-autonomous role for Notch2 in CNC cells. However, there is a lack of studies that look into how Notch manipulation contributes to CNC-derived heart development (37).

### 4.7 Multipotency of NCC

NCCs are multipotent, capable of differentiating into various cells in our bodies (18, 44). The multipotent capacity of the NCC are attributed to the combination of complex multimodal gene regulatory mechanism (44). Studies have shown that CNC could be differentiated into VSMCc, which could be employed for disease modelling and drug screening in relation to vasculopathies (17, 18).

Furthermore, second heart field (SHF) progenitors are multipotent cardiac progenitor cells that play an essential role with the cardiac NCCs in the development of the OFT and closely interact with each other (37). As such, SHF is responsible for forming the heart tube, myocardium, smooth muscle, and endothelial cells.

### 4.8 Cardiovascular neurocristopathy

Neurocristopathies are a group of genetic disorders that affect the development of cells derived from the NC. These could be from abnormalities in NCC migration, differentiation, or proliferation leading to structural abnormalities (45).

We have used the term cardiovascular neurocristopathy here to describe cardiopathy, aortopathy, and its related sequelae of the vascular and cardiac-specific anatomic region related to the CNC cell migration.

**Figure 5:**
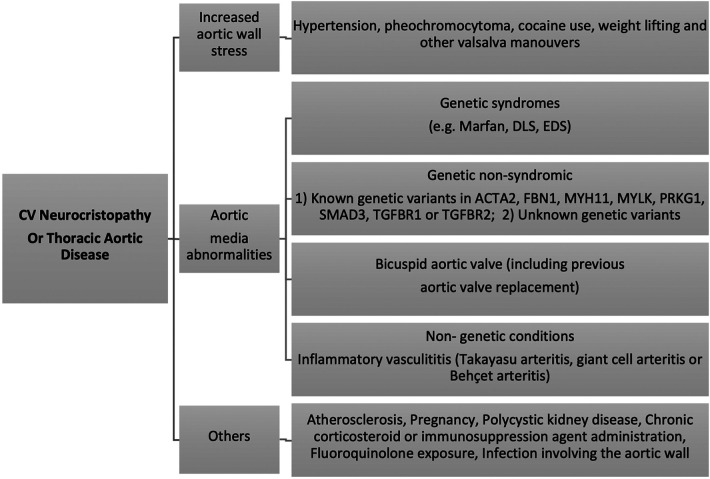
Cardiovascular neurocristopathy aetiologies and mechanisms.

Cardiovascular neurocristopathy occurs in the form of (1) cardiac septation disorders, mainly the aortico-pulmonary septum; (2) great vessels and vascular disorders; (3) myocardial dysfunction; and (4) a combination of all three phenotypes. This could be attributed to genetic, familial, sporadic or acquired causes (Figure 5) (5). For ease of understanding, we have divided the cardiovascular neurocristopathy into cardiac and vascular neurocristopathy.

Ablation studies have shown that NC abnormalities resulted in excitation-contraction (EC) coupling defects, especially contractility and L-type calcium current, causing abnormal myocardial function and death in-utero, resulting from cardiac failure (35, 36, 46). After CNC ablation, all the embryos showed abnormal myocardial function, mis-patterning of the arch arteries and glandular defects. In comparison, persistent truncus arteriosus was observed in 90%, while the remaining showed arterial pole misalignment defects, such as double-outlet right ventricles (5, 20, 35, 36, 46, 47).

#### 4.8.1 Cardiac neurocristopathy

A failure of CNC cells to migrate to the developing heart can result in cardiac neurocristopathy, including abnormality of cardiac septation, outflow tract obstruction, myocardial dysfunction, and heart rhythm abnormality (6).

The abnormal aorto-left ventricular interaction could lead to altered aortic dilatation and loss of elasticity, which can induce left ventricular hypertrophy, reduce coronary blood flow, and eventually cause left ventricular failure (5, 6, 11). However, the most important clinical phenotype of cardiac neurocristopathy is the BAV.

BAV, the most common congenital cardiovascular abnormality, affects 0.9%–2% of the general population (7, 48). It causes greater morbidity and mortality than all other congenital cardiovascular abnormalities combined.

BAV patients' VSMCs are less differentiated due to a phenotypic switch failure, which results in considerably decreased expression of differentiated, contractile VSMC markers such as smoothelin, calponin, and SM22alpha. Furthermore, lamina A/C, which is important for VSMC differentiation, is considerably expressed lower in the BAV population compared to the tricuspid aortic valves (TAV) population (9).

Furthermore, although many CHDs are considered non-syndromic, certain hereditary syndromes, like DiGeorge and CHARGE, entail NC deficits with established cardiac phenotypes (37). Not only do NC deficits cause cardiac disorders, but they are also known to contribute to a variety of craniofacial malformations that are frequent in human cardio-craniofacial syndromes, including the most common multiple anomaly syndromes, DiGeorge, Noonan, and Velo-cardio-facial (49, 50).

#### 4.8.2 Vascular neurocristopathy

Defects in NC migration and differentiation can also lead to abnormalities in the aortic arch and the great vessels of the head and neck, resulting in conditions such as coarctation of the aorta, interrupted aortic arch, and abnormalities in the origins of the brachiocephalic vessels (6, 51). This includes the thoracic aorta, innominate artery, its branches, left common carotid, left subclavian and OFT septae.

Aortic diseases involving the NC zone or vascular neurocristopathies could be categorized as (1) abnormal elasticity or increased aortic wall stress, (2) dilatation and aneurysm formation, and (3) dissection and rupture.

Vascular neurocristopathy constitutes aortic arch and carotid artery disorders, the most common phenotypes being the thoracic aortic aneurysm (TAA). As NC contributes to smooth muscle cells (SMCs) of the aortic arch, the innominate, and the right and left common carotid arteries, carotid artery dissection is other possible non-syndromic neurocristopathies. Additionally, defects in pericyte function can lead to weakened blood vessels, making them more prone to dissection. Pericytes in the aorta could have different origins, including SHF, neural crest, and somites (32, 33, 52, 53). Recent studies also suggest that gene mutations increase the risk of cervical carotid dissection. These mutations can affect the formation of the arterial wall, making it more susceptible to tearing (54).

Dilatation of the thoracic aorta could be due to intrinsic, hemodynamic abnormalities or both. Intrinsic here refers to the genetic theory, whereby the presence of aortic wall fragility is a consequence of a common developmental defect involving the aortic valve and the aortic wall (55, 56). TAA can be either hereditary or sporadic, the latter of which occurs without any known family history. It is estimated that genetic factors are responsible for about 25% of all TAA cases. Of this percentage, one-fifth are linked to a known genetic disorder, while the remaining patients with TAA have a family history of aneurysmal disease, but the specific gene causing it is yet unknown (54, 57).

One such condition is acute aortic syndrome (AAS), coined by Vilacosta et al. in 1998, which is a group of severe and potentially fatal aortic conditions, such as acute aortic dissection (AAD), traumatic aortic transection (TAT), intramural hematoma (IMH), penetrating aortic ulcers (PAU), and TAA post-SAD, all of which cause significant risk to life (58, 59).

Cervical carotid dissection and aberrant right subclavian artery are other such phenotypes linked with NCC, however, its exact cause is not yet fully understood (60). During embryonic development, the NCC migrate and differentiate into smooth muscle cells, which form the arterial wall, and pericytes, which support the developing blood vessels (32). Cardiac fibroblasts are derived from heterogeneous source, including the hematopoietic system, endothelium, epicardium, and neural crest (61).

Abnormalities in NCC migration, differentiation, or proliferation could lead to structural abnormalities in the arterial wall, making it more susceptible to tearing. Additionally, defects in pericyte function can lead to weakened blood vessels, making them more prone to dissection (32).

An aberrant right subclavian artery is also associated with abnormal NC migration. When the right fourth pharyngeal arch artery regresses improperly, it causes an aberrant right subclavian artery. In this situation, the right dorsal aorta remains abnormally cranial to the seventh intersegmental artery and creates the retroesophageal section of the right subclavian artery. This vascular abnormality is strongly linked to CHD affecting the outflow tract (OFT) (62).

#### 4.8.3 Co-occurrence of cardiac & vascular neurocristopathy

**Figure 6:**
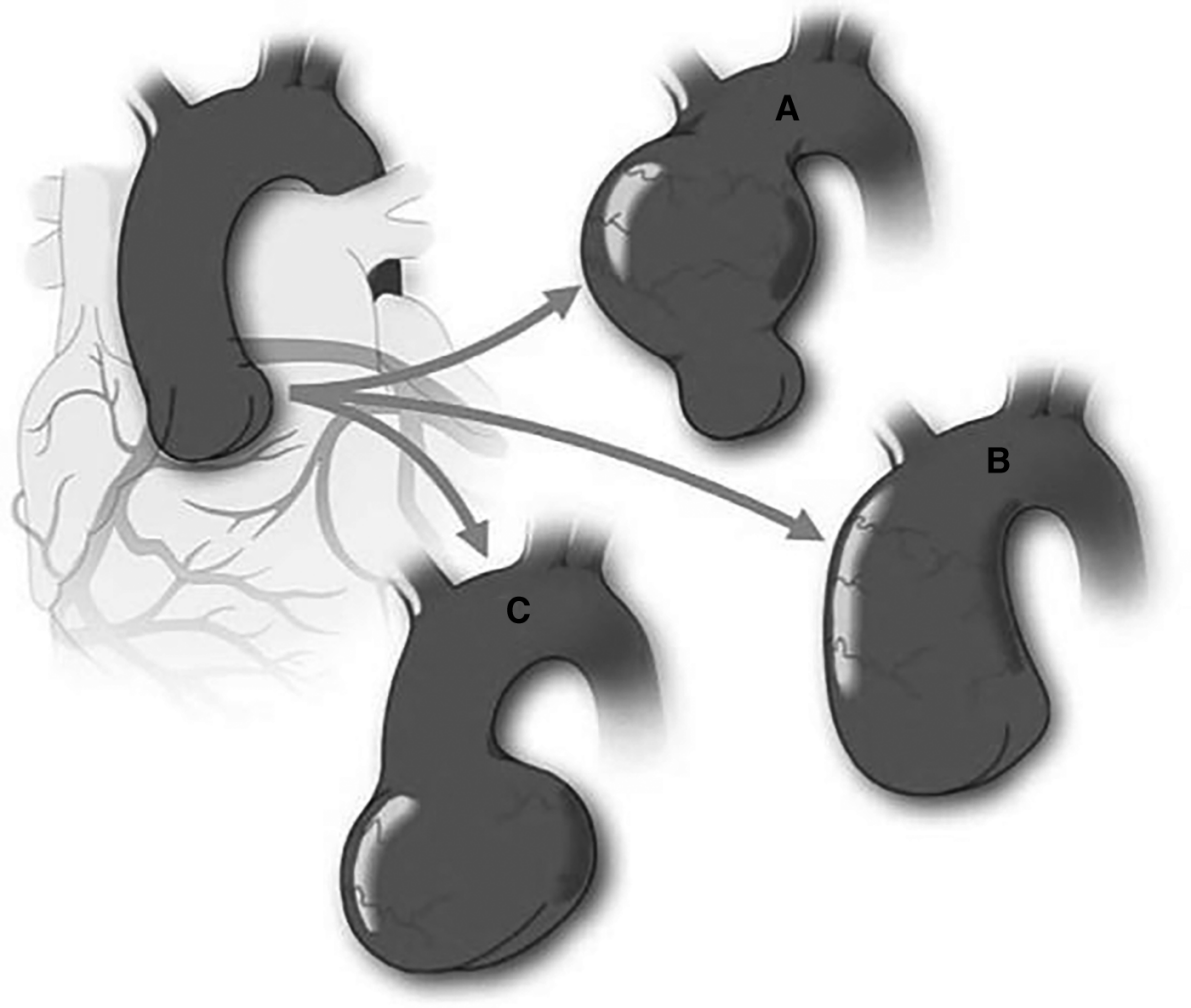
Schematic of variable aortic phenotypes encountered in bicuspid aortic valve (BAV). Figure demonstrates the different aortic dilatation patterns that may occur in BAV in comparison with a normal aorta (Top left). Although the most common portion to dilate is the tubular ascending aorta (**A**), the entire ascending aorta may be affected, including sinuses of Valsalva and tubular aorta with sinotubular junction effacement (**B**) There is a subgroup of BAV patients who exhibit dilatation of the sinuses of valsalva preferentially (**C**) This pattern is associated with type 1 (right-left fusion) BAV and male sex. Reprinted with permission from Michelena et al. (7). Copyrights 2023 Wolters Kluwer Health, Inc.

The semilunar valves' embryonic genesis is linked to the formation of the ascending aorta (25). During late gestation, the NC plays a crucial role in developing the outflow endocardial cushions, the precursors of the semilunar valves. The NC is responsible for remodelling and positioning the cushions, mesenchymal apoptosis, and proper valve architecture (63). Moreover, it also contributes to forming the smooth muscle layer wall of the ascending aorta and aortic arch. Therefore, any defects in the CNC can result in semilunar valve functional abnormalities and/or aortic arch artery abnormalities. Abnormalities in the process of NCC condensation at the early stages of outflow cushion formation may provide a common mechanism underlying BAV and explain the link between arterial wall anomalies and outflow malalignment defects. As a result, a disruption in the early developing pathways might result not only in a BAV but also in the accompanying aortopathy. Given this, BAV patients are at a significantly elevated risk of developing a thoracic aortic aneurysm (Figure 6) (64).

Patients with BAV might have medial abnormalities in the form of elastic fibre fragmentation and smooth muscle cell loss, previously termed “cystic medial necrosis”, which is like Marfan syndrome (65). However, the flow abnormalities associated with BAV may contribute to the aortopathy and aortic dilatation (9). TAAs form in roughly 50%–70% of BAV patients, much greater than the incidence of TAA generation in patients with tricuspid aortic valves (TAV) (66).

Aortic dilatation occurs early in the BAV person and is often characterized by mid-ascending dilation. It is still uncertain whether aneurysms in BAV patients are caused by changed hemodynamic forces caused by the defective valve or by an underlying genetic flaw that causes both BAV and TAA (48). The evaluation of hemodynamic flow in BAV patients reveals major changes in flow patterns compared to TAV patients (67).

As BAV is a heterogeneous disease with different aortic phenotypes, any decision to extend a proximal aortic repair into the arch while repairing the BAV valve must be individualized. Small subsets of patients may benefit from prophylactic hemiarch resection during proximal repair. However, it is challenging to identify those patients who could potentially develop proximal arch disease.

Furthermore, people with BAV have an eight-fold increased risk of aortic dissection (68). Despite aortic valve replacement to improve hemodynamics, some BAV patients develop an ascending aortic aneurysm (69). Using aorta tissue from BAV patients, researchers discovered possible indications of aortopathy of the aortic wall, including immature SMCs (9). Aneurysms frequently include the aortic root, ascending aorta, and aortic arch, although the descending aorta is rarely compromised in BAV/TAA (10).

Congenital BAV is the first nonsyndromic CHD where aortic dissection and dilation are documented (21). Aortic dissection is nine times more common in BAV patients than in tricuspid aortic valve patients and presents at a relatively younger age (55 vs. 63 years) (57). In BAV patients, aortic dilatation starts in childhood, independent of aortic regurgitation or aortic stenosis (70).

Histopathological studies of non- and dilated ascending aortic wall samples from BAV and TAV patients have shown several structural discrepancies in recent years. Larson et al. reported one of the first studies that revealed variations in the ascending aorta of BAV and TAV in 1984 (71). Vascular smooth muscle cells (VSMCs) play an important role in distinguishing BAV from TAV histology. VSMCs are not only detected in apoptosis, but they are also physically different in the BAV population (72).

### 4.9 CNC ablation phenotypes

The significance of CNC cells in heart development and function has come from research on chick embryos after the pre-migratory CNC was removed (11). This NC ablation paradigm was the first reliable animal experimental model of congenital heart abnormalities and has served as the “gold” standard for characterizing the aetiology of heart problems in later experimental models, including transgenic mice (24).

The major component of the CNC ablation phenotype is myocardial function abnormalities. Myocardial dysfunction, like the looping defect, is initially noticed in NC-ablated embryos around the time NCC should migrate into the caudal pharyngeal arches, many days before the typical entry of NCC into the outflow canal (73). CNC ablation also results in altered SHF proliferation and abnormal myocardial function as secondary effects (25). Following CNC ablation, abnormal looping is the earliest visible defect observed, even before CNC cells reach the OFT. Furthermore, defective looping could also be seen if the OFT myocardium fails to add to the heart tube from the anterior subpopulation of SHF cells. Thus, the looping defects observed after CNC ablation suggest that CNC cells are required for the normal deployment of SHF cells. Altered looping could also lead to hemodynamic changes, potentially causing flow related pathologies (74).

These embryos adjust for reduced contractility by dilatation of the ventricles, allowing them to retain enough cardiac output for survival (75). These functional compensations during very early cardiac development are hypothesized to play an etiologic role in the eventual development of structural heart abnormalities. At older ages, when septation would ordinarily be complete, these embryos have a lower ejection percentage and poor contractility in the myocardium (49). Reduced embryo weight and oedema also suggest poor cardiac function (76).

CNC ablation also results in altered SHF proliferation and abnormal myocardial function as secondary effects (77). Abnormal looping is the earliest defect seen after CNC ablation and can be observed before the CNC cells reach the OFT. Defective looping may be caused by the failure of the addition of the OFT myocardium to the heart tube from the anterior subpopulation of SHF cells. Thus, the looping defects observed after CNC ablation suggest that CNC cells are required for the normal deployment of SHF cells (78).

### 4.10 Scrutiny of the systematic review for 40 articles

While this systematic review has endeavoured to shed light on the complex realm of neurocristopathy, it is important to acknowledge its inherent limitations. The vast heterogeneity observed in the 40 manuscripts analyzed underscores the intricate and multifaceted nature of this field, revealing that the science surrounding neurocristopathy is still in its infancy. Here, we outline the key limitations of this review:

### 4.11 Heterogeneity of data

The foremost limitation arises from the remarkable heterogeneity observed within the selected manuscripts. The diversity in study design, patient populations, and research methodologies across the included articles made it challenging to conduct a uniform analysis. This heterogeneity emphasizes the need for standardized approaches in future investigations in neurocristopathies.

### 4.12 Limited sample size

Many of the manuscripts under review presented data from relatively small sample sizes. This limitation raises questions about the generalizability of the findings and underscores the need for larger and more comprehensive studies to draw definitive conclusions.

### 4.13 Heterogeneity in definitions and diagnostic criteria

The lack of universally accepted definitions and diagnostic criteria for neurocristopathy-related conditions posed a significant challenge. This variability in terminology and classification across studies hinders efforts to consolidate and compare data accurately.

### 4.14 Data quality and reporting

The overall quality of data and reporting in some of the included manuscripts was variable. Incomplete data, vague reporting of methods, and limited statistical analyses in certain articles hindered our ability to perform a robust and consistent review.

### 4.15 Publication bias

Systematic reviews are inherently susceptible to publication bias, where positive findings are more likely to be published than negative or inconclusive ones. This bias may have influenced the selection of studies included in this review, potentially skewing the overall findings.

### 4.16 Inherent challenges of a nascent field

The review's limitations are compounded by the fact that the field of neurocristopathy is still in its infancy. The limited body of research and the evolving understanding of the subject matter pose inherent challenges in conducting a comprehensive review.

### 4.17 Lack of longitudinal data

Many of the manuscripts presented cross-sectional data, lacking longitudinal follow-up of patients. Long-term outcomes and the natural history of neurocristopathy-related conditions remain areas of uncertainty.

### 4.18 Language and geographic bias

The restriction to articles published in the English language may introduce language bias, potentially omitting relevant research published in other languages.

As the science surrounding neurocristopathy is still evolving and much work remains to be done, addressing these limitations requires collaborative efforts from the scientific community to standardize definitions, conduct larger and more comprehensive studies, and foster a global and multidisciplinary approach to advancing our understanding. Despite the challenges, this systematic review represents a critical step in the ongoing journey to unravel the mysteries of neurocristopathy and pave the way for more effective diagnosis and treatment in the future.

## 5 Discussion

The traditional perspective on atherosclerosis as a leading player in the development of aortic aneurysms and dissections has been challenged by emerging research. While the well-established link between atherosclerosis and these conditions cannot be denied, recent studies have introduced complexity to this narrative. These studies raise questions about the multifactorial nature of aortopathy, suggesting that the causes might extend beyond atherosclerosis (79).

Herein, we contemplate the role of CNC cells and other factors, such as the gut microbiome and intrinsic inflammatory processes, in shaping the landscape of aortopathy. This evolving understanding urges us to explore the intricate web of interconnected factors that contribute to the pathogenesis of aortopathy (80).

Of paramount importance is the role played by CNC cells in the genesis of CHDs. As our ability to screen and detect CHDs in childhood has improved, we have witnessed a rise in the recognition of CNC deficit phenotypes associated with CHDs. This observation underscores the interplay between aortopathy and cardiac abnormalities and prompts the need for further research to identify the underlying pathogenic factors and genetic deficits responsible for these cardiac anomalies. Such discoveries are essential for enhancing diagnostic screening and refining therapeutic interventions, ultimately improving the lives of individuals affected by these conditions (57, 81).

Phenotypes characteristic of cardiovascular neurocristopathy, such as BAV and TAA, share a common embryonic origin in CNC cells and are surprisingly prevalent in the general population. BAV, with or without associated aortopathy, affects around 2% of the population, while TAA is observed in 1% of individuals. Within this realm, TAA encompasses hereditary and sporadic cases, with an estimated 25% of all TAA cases having a genetic component (7, 48).

About one-fifth of TAA cases are linked to known genetic disorders, while the remaining cases have a positive family history of the condition, albeit with an unknown causative gene (70). This complex landscape emphasizes the need for a deeper understanding of thoracic aorta development and both normal and defective aortic valves (6). In particular, it is critical, given the lack of available medications to prevent TAA in BAV patients.

The development of aortopathies, including aneurysms and dissections, is a potentially life-threatening process, fraught with the lifelong risk of catastrophic aortic events. Detecting thoracic aortic aneurysms early and preventing acute aortic syndromes is a formidable challenge.

Historically, formal screening of aortic diameter has been the primary method for identifying these conditions and preventing life-threatening complications. However, the increasing incidence of potentially fatal aortopathies, such as acute aortic syndromes, demands a more profound understanding of aortic pathologies at the cellular level and their genetic underpinnings. This knowledge is essential for anticipating the likelihood of aortic complications in specific patients and tailoring early preventive and therapeutic interventions to reduce cardiovascular morbidity and mortality (57).

In essence, a better grasp of cardiovascular neurocristopathies paves the way for a deeper understanding of disease pathophysiology, enabling the formulation of precision management strategies for these potentially fatal cardiovascular conditions. This journey towards precision medicine is one that holds the promise of better outcomes for those grappling with aortopathies and related cardiac anomalies.

### 5.1 Future perspective

It is imperative to gain a precise understanding of the ultimate phenotype of central cardiovascular structures. This understanding will pave the way for the separation of processes contributing to this final phenotype. By defining novel models of cardiovascular dysmorphogenesis, we can explore interactions of structure and function, a dynamic relationship that ultimately shapes the phenotype. This journey will enable us to comprehend the involvement of specific genes in the early stages of dysmorphogenesis and the factors influencing cardiovascular patterning.

Molecular research in cardiovascular neurocristopathy represents a pivotal moment in our understanding of cardiovascular health, offering the promise of insights into the intricate mechanisms guiding the formation of the heart.

Genetic abnormalities associated with aortopathies underscore the critical role played by smooth muscle cells and the extracellular matrix in shaping the destiny of cardiovascular neurocristopathies. These revelations provide a glimpse into cardiovascular system, shedding light on the genetic underpinnings of these conditions.

Furthermore, the highly heritable nature of aortic size and elasticity points to the potential role of multiomics and polygenic risk scores in the early detection of sporadic cases of cardiovascular neurocristopathies. This promising avenue could revolutionize our approach to screening and diagnosis, enabling early intervention and personalized management strategies.

Molecular insights, genetic revelations, and advanced screening techniques converge to offer a brighter and healthier future for those who contend with these intricate cardiovascular conditions. This exciting period in heart development promises to reshape the landscape of cardiovascular medicine and enhance our ability to prevent, diagnose, and treat these challenging disorders.

## 6 Conclusion

Cardiovascular neurocristopathy emerges as a possible instigator of a spectrum of cardiac and vascular aortopathies, calling into question the very foundations of our understanding of these conditions. To navigate this uncharted territory and pave the way for a brighter, healthier future for those who grapple with these intricate cardiovascular anomalies, we advocate for a concerted effort in preclinical and clinical research.

In the realm of cardiovascular health, the enigmatic world of neurocristopathies unfolds as a potential harbinger of thoracic aneurysms, dissections, and the catastrophic rupture of life's vital conduits. It beckons us to explore the uncharted territories of the heart and vascular system, revealing a panorama of pathologies that remain largely unaddressed by conventional disease paradigms. By delving into the differentiation of CNC cells, we can unlock the mysteries of cardiac neurocristopathies. This knowledge holds the potential to reshape our approach to diagnosis, treatment, and prevention, offering a lifeline to those at risk of these devastating cardiovascular events.

The cardiovascular community is poised to rewrite the narrative of aortic pathologies and cardiac dysmorphogenesis. The quest for a deeper understanding of CNC cells and their role in cardiac neurocristopathies represents a beacon of hope, promising to illuminate the path toward better cardiovascular health and a future where these potentially life-threatening conditions are conquered.

## Data Availability

The raw data supporting the conclusions of this article will be made available by the authors, without undue reservation.
